# Prescription Drug Misuse in “Clubbers” and Disco Goers in Ibiza

**DOI:** 10.3389/fpsyt.2020.592594

**Published:** 2020-12-15

**Authors:** Massimo di Giannantonio, Attilio Negri, Stefania Schiavone, Chiara Vannini, Mauro Pettorruso, Fabio De-Giorgio, Valeria Verrastro, Luigia Trabace, Mariangela Corbo, Rossella Gottardo, Cristian Camuto, Monica Mazzarino, Andrea Barra, Domenico De Berardis, Juan Iglesias Lopez, Cristina Merino Del Villar, Fabrizio Schifano, Giovanni Martinotti

**Affiliations:** ^1^Department of Neuroscience, Imaging, Clinical Sciences, University G.d'Annunzio, Chieti-Pescara, Italy; ^2^Department of Clinical, Pharmaceutical and Biological Sciences, School of Life & Medical Sciences, University of Hertfordshire, Hatfield, United Kingdom; ^3^Postgraduate School of Clinical Pharmacology and Toxicology, University of Milan, Milan, Italy; ^4^Department of Clinical and Experimental Medicine, University of Foggia, Foggia, Italy; ^5^Department of Health Care Surveillance and Bioethics, Section of Legal Medicine, University Cattolica del Sacro Cuore, Rome, Italy; ^6^Fondazione Policlinico Universitario A. Gemelli IRCCS, Rome, Italy; ^7^Department of Medical and Surgical Sciences, Magna Graecia University of Catanzaro, Catanzaro, Italy; ^8^Unit of Forensic Medicine, Department of Diagnostics and Public Health, University of Verona, Verona, Italy; ^9^Laboratorio Antidoping FMSI, Rome, Italy; ^10^Azienda Sanitaria Locale Potenza, Potenza, Italy; ^11^NHS, Department of Mental Health, Psychiatric Service for Diagnosis and Treatment, Hospital “G. Mazzini,” ASL 4, Teramo, Italy; ^12^Can Misses Hospital, Ibiza, Spain; ^13^Psychopharmacology, Drug Misuse & Novel Psychoactive Substances Research Unit, School of Life & Medical Sciences, University of Hertfordshire, Hatfield, United Kingdom

**Keywords:** prescription drugs, novel psychoactive substance (NPS), club drugs, psychopathology, substance usage disorders (SUDs)

## Abstract

**Background:** Prescription drug misuse and its related risks are considered a worldwide public health issue. Current trends show that the extent of such phenomenon may not be limited to subjects with psychiatric disorders, as it also spreads to dance party and nightclub attendees, who often consume prescription drugs in combination with alcohol and psychoactive substances. This study aims to report the sociodemographic data and the psychiatric and clinical features of a sample of clubbers reporting prescription drugs use.

**Methods:** Patients admitted to the psychiatry ward of the Can Misses Hospital in Ibiza were recruited for the study during a span of four consecutive years (2015–2018). The inclusion criteria were age 18–75 years old and the intake of psychoactive substances or more than five alcohol units during the previous 24 h. Substance use habits, psychopathological features, and use of unprescribed pharmaceuticals were investigated. Urine samples were collected and analyzed using gas chromatography/mass spectrometry.

**Results:** A total of 110 subjects with psychoactive substance intoxication were recruited for the study. Among these, 37 (40%) disclosed the use of prescription drugs without medical supervision. The most common compounds were benzodiazepines (66%), antiepileptic drugs (8%), antidepressants (6%), opioids (6%), antipsychotics (6%), stimulants (6%), and non-steroidal anti-inflammatory drugs (NSAIDs, 2%). Prescription drug misuse was negatively associated with the use of psychodysleptics (two-tailed Fisher's exact test *p* = 0.018, ρ = −0.262).

**Conclusions:** The use of prescription drugs is also common among clubbers, usually characterized by low propensity to be prescribed benzodiazepines, antipsychotics, or antidepressants. Prescription drugs may be an alternative to classic and novel psychoactive compounds or may be used to tamper and self-medicate the effects determined by the use of substances. Party goers should be adequately informed about possible risks of co-intake of psychoactive substances and prescription drugs to prevent serious medical and psychiatric consequences.

## Background

Prescription drug misuse and related risks, including co-ingestion with recreational drugs, have recently risen as a worldwide public health phenomenon. They may involve a variety of medical and social consequences that require effective public health policies to counteract such habit, as well as continuous updates for health professionals to promote education and harm reduction (1, 2). Prescription medicine misuse or non-medical use is commonly defined as the use of medications without a prescription or in a manner other than prescribed (3). This includes a number of conditions, such as using these compounds for purposes other than the medical condition they were prescribed for (i.e., recreational use or self-harm), consuming at larger doses or higher frequencies than intended, using an alternative route of administration (e.g., intravenous), or co-using with alcohol or recreational drugs (4). Studies report that the prevalence of misuse of any prescription drug in the United States increased by 67% from 1991–1992 to 2001–2002, while treatment-seeking for prescription drug use disorders increased by 53% (2). In 2017, 14 countries in EU reported on the non-medical use of such compounds (5). Among the 10,956 drug-related acute toxicity emergency room (ER) presentations in the Euro-DEN Plus dataset, approximately 29% involved at least one prescription medicine (most commonly benzodiazepines and opioids), and 45% of these involved only prescription drugs, with no illicit compounds involved (6).

Current trends show that the extent of prescription drug misuse is not limited to subjects with psychiatric disorders or co-occurring substance use disorders (SUDs). Admissions to ER and psychiatric intensive care units due to psychotropic pharmaceutical intoxications involve a heterogeneous cohort of users, including traditional drug users, “psychonauts” [from the Ancient Greek ψ υ χ ή (soul) and ν α ύ τ η ς (sailor), i.e., subjects who define themselves as explorers of the human soul through the use of psychoactive substances], clubbers, students, marginalized populations, and individuals with patterns of non-habitual recreational drug consumption (7). In this context, the phenomenon of co-ingesting prescription drugs in order to imitate, potentiate, modulate, or counteract the effects of prohibited psychoactive substances has been increasingly reported (8). This trend involves not only novel highly potent opioid, such as fentanyl and its derivatives, or designer benzodiazepines but also antipsychotics, antidepressants, stimulants, performance-enhancing drugs (PEDs), hormones, vitamins, beta-blockers, gabapentinoids and over-the-counter (OTC) drugs (8).

For example, students and workers may consume attention deficit hyperactivity disorder (ADHD) medications such as methylphenidate to improve their academic performance or working tasks (1). Gamma hydroxybutyrate (GHB), a drug used for many conditions, has been increasingly associated with practices such as “chemsex” (9). Furthermore, compounds such as benzodiazepines (e.g., diazepam and alprazolam) or atypical antipsychotics (e.g., quetiapine and risperidone) are often used by club goers to counteract the effects of psychostimulant drugs, such as cocaine or methylenedioxymethamphetamine (MDMA) (10, 11). Venlafaxine, a selective noradrenaline reuptake inhibitor, has been associated with recreational use at high dosages, earning for itself the name of “baby ecstasy” (i.e., MDMA) (8). With regard to the nightlife and clubbing scene, the situation shows peculiar characteristics. The growing offer of novel and traditional prescription drugs has found a fertile ground in this scenario. Summer holiday periods in popular resorts have historically represented an opportunity for excesses and experimentation, especially among young people who find an environment in which hedonistic partying is socially accepted and drugs are typically easily available (12). Alcohol use, particularly during binge drinking, and psychoactive substance use are commonly reported among festival-goers and clubbers in holiday resorts; practices such as poly-substance abuse and prescription drug misuse have also been reported (13–16). The use of a variety of pharmaceuticals including benzodiazepines (17, 18), stimulants (19, 20), opioids (21), antidepressants (8), and sedatives such as GHB (22) has been associated to dance music party attendees. Such heterogeneous cohort of compounds, presented in different forms and with various ways of intake (e.g., ingested, snorted, or intravenous), may lead to potential negative medical outcomes, including acute intoxications, SUD, and other psychiatric disorders. Nevertheless, pharmaceuticals are often perceived as less harmful and less stigmatizing than illicit drugs, particularly among young people, partly due to these substances' legitimate medical purposes (23, 24). Moreover, information on the actions of these drugs is widely available in package inserts, advertisements, and on the internet; therefore, their effects (including adverse reactions) and dosages are considered more predictable (25).

Such phenomenon is further complicated by the rise on the nightlife market of novel psychoactive substances (NPS). A number of these substances were originally developed as research chemicals and diverted for recreational purposes, as they often mimic the pharmacological effect of traditional drugs of abuse or popular prescription drugs (4). Their effects and related risks are often unknown to both users and health professionals, due to the scarcity of evidence-based information regarding their toxicological profiles and to the ever-changing nature of this market (7, 26–28). Nevertheless, growing evidence reported potential acute and chronic psychiatric risks associated to NPS consumption, including confusion; paranoid thoughts; auditory and visual hallucinations; dissociation; delusions of reference, persecution, grandeur, and jealousy; cognitive impairment; hypomanic states; aggressiveness and irritability; violence; and suicidal thoughts (8, 29–31).

The current dynamic of recreational substance use is a serious matter of concern for public health institutions worldwide. In particular, the threats posed by psychoactive compounds and concomitant prescription drug misuse require updated policies provided by local and supranational regulatory agencies, as well as appropriate approaches by health professional, to prevent negative outcomes and reduce associated harms (32), including deaths (33). In such context, Ibiza and the Balearic Islands, two of the most popular destinations with nightlife resorts for summer holidays in Europe, may be considered as an interesting real-life scenario to explore such phenomenon. Previous studies confirmed a higher prevalence of risky behaviors for both residents and tourists in Ibiza, including problematic alcohol use, substance use, and sexual disinhibition (34–36). Moreover, it has been reported that traffickers and dealers have introduced NPS and pharmaceuticals into the Ibiza drug market to test new compounds and drug combinations on unaware customers (36).

This study aimed to assess patients admitted to the psychiatric ward of the Can Misses Hospital in Ibiza for psychoactive substance intoxication, in order to (1) identify which psychotropic prescription drugs are mostly involved in cases of concomitant psychoactive substance use and (2) report the psychopathological features and patterns of consumption associated to prescription drug use in a nightlife resort setting.

## Materials and Methods

Patients admitted to the psychiatry ward of the Can Misses Hospital in Ibiza during summer when nightclubs are open (May–October) were recruited for the study during a span of four consecutive years (2015–2018). The subjects were evaluated according to the DSM-5 diagnostic classification. The inclusion criteria were age 18–75 years old and the intake of psychoactive substances or more than five alcohol units (i.e., 10 ml or 8 g of pure alcohol) during the previous 24 h. Clinical conditions such as *delirium tremens*, epilepsy, liver encephalopathy, dementia, and other neurological diseases, severe cardiac failure, diabetes mellitus, severe liver impairment, kidney failure, or neoplastic diseases were among the exclusion criteria, as the presence of such conditions could present a confounding factor. Demographic (age, gender, family, and nationality) and socioeconomic data (living status, job status, and level of education) were collected, as well as recent and past medical and psychiatric history, current pharmacological treatment, and alcohol and substance use habits (including NPS), with a specific focus on prescription drugs misuse. Among these, recent and lifetime use of benzodiazepines (e.g., diazepam, alprazolam, and lorazepam), ADHD medications (e.g., amphetamine/dextroamphetamine and methylphenidate), and opioid painkillers (e.g., morphine, methadone, oxycodone, and fentanyl), as well as other popular prescription drugs (e.g., GHB and gabapentinoids) was investigated.

To explore the different psychopathological aspects related to substance use, such as depressive or manic symptoms, anxiety, psychosis negative and positive symptoms, somatic disorders, aggressiveness, and suicidality, the following psychodiagnostic tests were administered to patients during their hospitalization: Timeline Follow-Back (TLFB) for psychoactive substances and alcohol; Brief Psychiatric Rating Scale (BPRS); Positive and Negative Symptoms Scale (PANSS); Mania Rating Scale (MRS); Hamilton Depression Scale (HAM-D); Hamilton Anxiety Scale (HAM-A); and Modified Overt Aggression Scale (MOAS). TLFB was used to identify the main substance of abuse for each patient. The subjects were divided in three macro-groups according to the TLFB and the results of the urinalysis: psychostimulants (e.g., cocaine, amphetamines, and synthetic cathinones), depressors (e.g., opioids, alcohol, and benzodiazepines), and psychodysleptics (e.g., cannabinoids, psychedelics, and dissociatives). This classification was derived from our previous reports on the topic (7, 36).

Data collection was carried out in an anonymous and confidential way; all participants received a detailed explanation of the design of the study and a written informed consent was systematically obtained from every subject, according to the Declaration of Helsinki. Ethics approval was granted by the University of Hertfordshire Health and Human Sciences ECDA, protocol no. aPHAEC1042(03); by the CEI Illes Balears, protocol no. IB 2561/15 PI; and by the University “G. d'Annunzio” of Chieti-Pescara, no. 7/09-04-2015. Majorcan local ethics committee also gave approval to the study.

### Urine Sample Analysis

A urine sample was collected at admission, stored at −30°C, and subsequently analyzed at the laboratory of the Department of Forensic Toxicology of the Università Politecnica delle Marche, at the FMSI Antidoping of Rome, and at the University of Verona, Italy. The urine samples were analyzed at the FMSI Antidoping of Rome using a routine screening test for drugs of abuse. The urine samples were extracted with a solid-phase cartridge (Oasis MCX), and the obtained solution was evaporated until dry and reconstituted with mobile phase. An Agilent 1290 Infinity II UHPLC with a binary gradient system and an automatic injector (Agilent Technologies, Cernusco sul Naviglio, Milano, Italy) was used for the chromatographic separation. The instrument was equipped with an Agilent ZORBAX Eclipse Plus C18 column (100 × 2.1 mm i.d., particle size 1.8 μm) (37). The detector was an Orbitrap Q Exactive (Thermo Fisher Scientific) with an ESI source. The method was validated according to WADA guidelines and for a screening method in antidoping test defining selectivity, limit of detection (LOD), recovery, carry over, and repeatability (38). The method showed no interference or carry over, LOD < 1 ng/ml, recovery >70%, and repeatability estimated as CV% < 1% for all the analytes.

A comprehensive screening of urine samples was performed at both the Unit of Forensic Medicine of the University of Verona and at the Politecnico of Ancona, by using a Toxtyper™ LC/IT-MS platform (Bruker Daltonics, Bremen, Germany) consisting of an ultra high performance liquid chromatography (UHPLC) coupled to a high-speed ion trap mass analyzer (IT-MS). The instrument applied the analytical protocols provided by the manufacturer, and compound identification was provided by using the Maurer/Wissenbach/Weber (MWW) library containing as many as 4,500 therapeutic, toxic/illicit drugs and their metabolites (including NPS) (39). Prior to injection, urine sample were diluted 1/10 (v/v) with water (40).

### Data Analysis

Statistical analysis was performed by using IBM SPSS® Statistics software, version 20 and GraphPad 5.0 software for Windows (La Jolla, CA, USA). Fisher's exact test was used to determine whether or not there was a significant association between the categorical variables “abuse of prescription drugs” and “use of distinct categories of psychoactive substances.” Spearman's correlation value (ρ) was calculated to determine if variables (abuse of prescription drugs and categories of substances) were positively or negatively correlated. Independent samples *t*-test was used to determine whether or not there was a significant difference in scale scores between subjects who abused and subjects who did not abuse prescription drugs. One-way analysis of variance (ANOVA) followed by Tukey's *post-hoc* test was used to assess whether or not there was a significant difference in scale scores among subjects who abused different classes of prescription drugs. For all tests, a two-tailed *p*-value <0.05 was considered statistically significant.

## Results

A total of 110 subjects were recruited for the study, with most of them being of European nationality (*n* = 76, 71.8%). Age ranged from 19 to 63 years old, with the majority of patients (*n* = 57, 51.8%) under 30 years old. The median age of the 110 patients was 32.57 years. A higher percentage of males (*n* = 76, 69.1%) was reported in our sample. Nine patients were full-time or part-time students (8.1%), 52 (47.3%) were employees, and 40 (36.4%) were unemployed.

All the subjects of the sample were diagnosed with substance intoxication at admission. Although the majority of patients declared multiple substance use (*n* = 77, 70.0%) and 33% of them reported the use of more than two substance, the participants were divided in three macro-groups according to their responses to the TLFB test and their urinalysis results to identify a category of substances “of choice” for each patient. Thus, 17 (15%) depressors users, 44 (40%) stimulant users, and 49 (45%) psychodysleptics users were identified.

When asked about lifetime use of specific groups of substances, stimulant use was disclosed by 74 patients (32%) and cannabinoid use by 68 patients (29%). These were followed by depressors (*n* = 32, 14%), empathogens–entactogens (*n* = 28, 12%), dissociatives (*n* = 15, 6%), opioids (*n* = 9, 4%), and psychedelic drugs (*n* = 7, 3%). Almost half of the participants (46%) declared to have used a substance without knowing what it was at least once in their life. These results will be described in a separate manuscript (31).

In our sample, 37 patients (40%) disclosed a lifetime misuse of prescription drugs. The most commonly reported compounds were benzodiazepines, which were used by 32 subjects. Table 1 presents the complete information on the type of pharmaceuticals reported by users.

**Table 1:** The most common substances used by patients who declared prescription drug misuse.

**Prescription Drug**	***N***	**%**
Benzodiazepines (e.g., diazepam and alprazolam)	32	66
NSAIDs (e.g., paracetamol)	1	2
Antidepressants (e.g., paroxetine and clomipramine)	3	6
Antipsychotics (e.g., risperidone and clotiapine)	3	6
Anticonvulsants (e.g., valproate and pregabalin)	4	8
Opioid derivatives and synthetic opioids (e.g., methadone and fentanyl)	3	6
Stimulants (e.g., methylphenidate)	3	6

**Figure 1:**
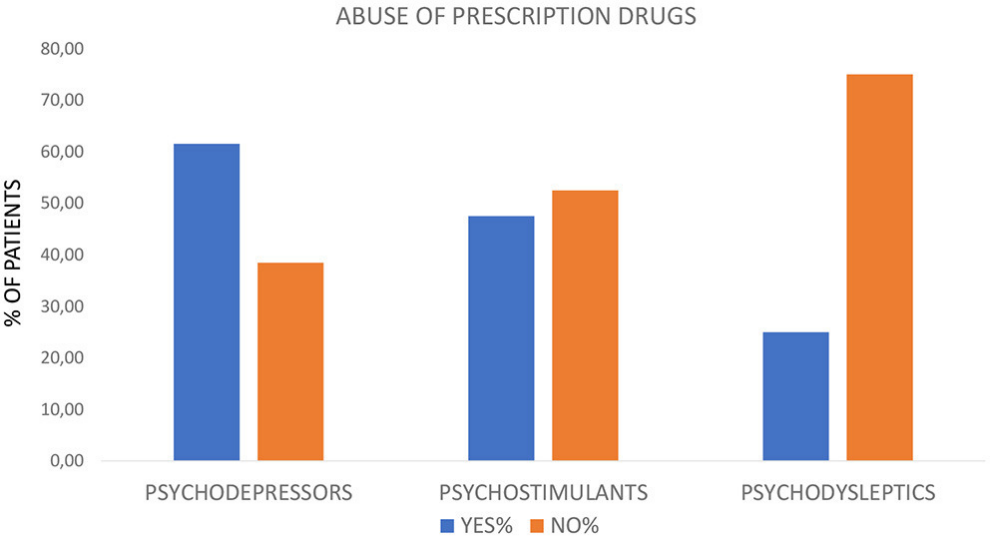
Percentage of patients abusing (Yes%) or not abusing (No%) prescription drugs stratified for the following substance categories: psychodepressors, psychostimulants, or psychodysleptics.

Prescription drug misuse was reported for 8 psychodepressor (e.g., non-prescription opioids and alcohol) users, 19 psychostimulant (e.g., cocaine and amphetamines) users, and 10 psychodysleptic (e.g., cannabis and dissociatives) users. The percentage for each group of substance users is reported in Figure 1. Abuse of unprescribed pharmaceuticals was negatively associated with the use of psychodysleptics (two-tailed Fisher's exact test *p* = 0.018, ρ = −0.262).

According to their lifetime use of specific compounds, prescription drug consumption without medical supervision was reported by 31 stimulant users, 21 cannabinoid users, 10 depressor users, 7 opioid users, 7 empathogen–entactogen users, 5 dissociative users, and 1 psychedelic user.

**Figure 2:**
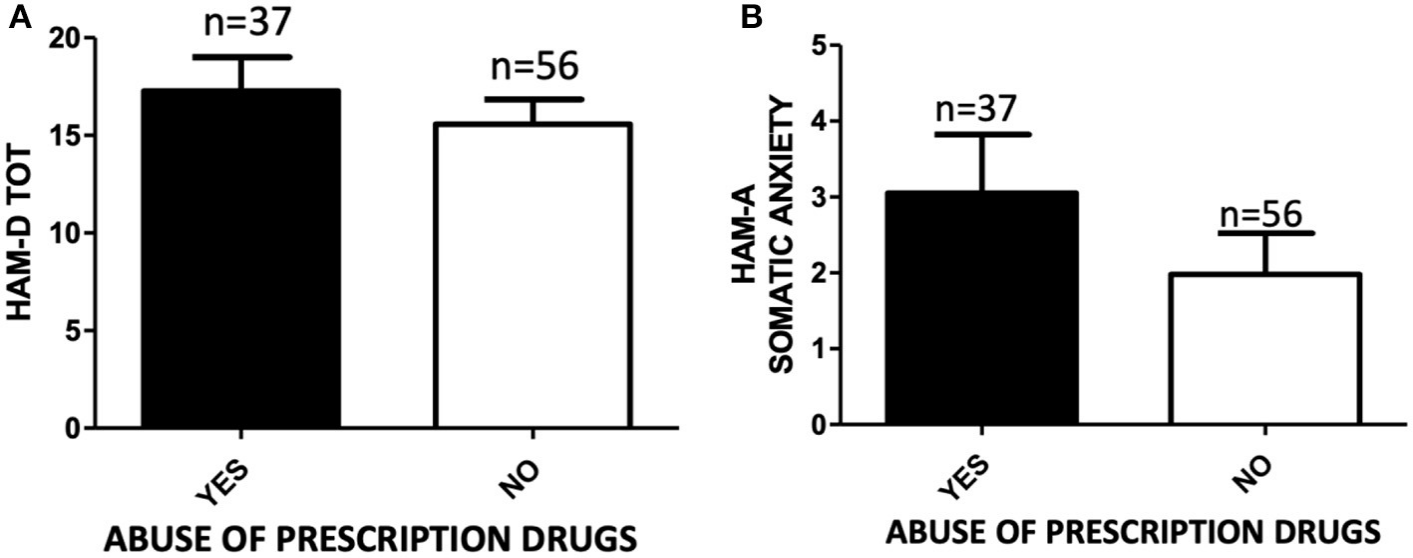
**(A)** HAM-D total score of subjects abusing (YES, *n* = 37) or not abusing (NO, *n* = 56) prescription drugs. Independent samples *t*-test, *p* > 0.05; **(B)** HAM-A Somatic Anxiety scale score of subjects abusing (YES, *n* = 37) or not abusing (NO, *n* = 56) prescription drugs. Student's *t*-test, *p* > 0.05.

The severity of psychiatric symptoms according to HAM-A Psychotic Anxiety scale, PANNS BPRS, and MRS was comparable among users and non-users of unprescribed pharmaceuticals. Patients who disclosed prescription drug misuse tended to report higher scores in HAM-D and HAM-A Somatic Anxiety, although this tendency did not reach the statistical significance (Figure 2).

**Figure 3:**
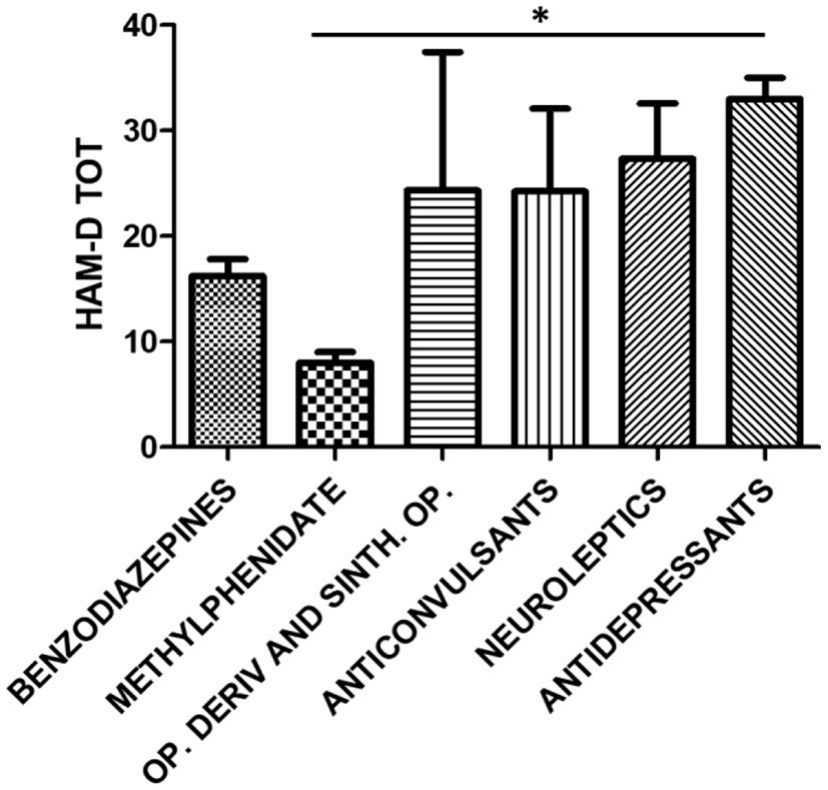
HAM-D total score stratified for the following classes of abused prescription drugs: benzodiazepines (*n* = 32), methylphenidate (*n* = 3), opioid derivatives and synthetic opioids (*n* = 3), anticonvulsants (*n* = 4), neuroleptics (*n* = 3), and antidepressants (*n* = 3). One-way ANOVA, followed by Tukey's *post-hoc* test, *F* = 3.032, *p < 0.05 methylphenidate vs antidepressants.

One-way ANOVA for HAM-A total score (*F* = 0.6808, *p* > 0.05), PANNS (*F* = 1.487, *p* > 0.05), MRS (*F* = 0.4402, *p* > 0.05), and BPRS (*F* = 3.094, *p* > 0.05) did not report any statistically significant difference among users of benzodiazepines, methylphenidate, prescription opioids, anticonvulsants, antipsychotics, and antidepressants. A statistical difference was found for HAM-D scores between methylphenidate and antidepressant users (one-way ANOVA, followed by Tukey's *post-hoc* test, *F* = 3.032, ^*^p < 0.05 methylphenidate vs. antidepressants) (Figure 3), with higher scores of depression in the group of patients taking antidepressants.

**Figure 4:**
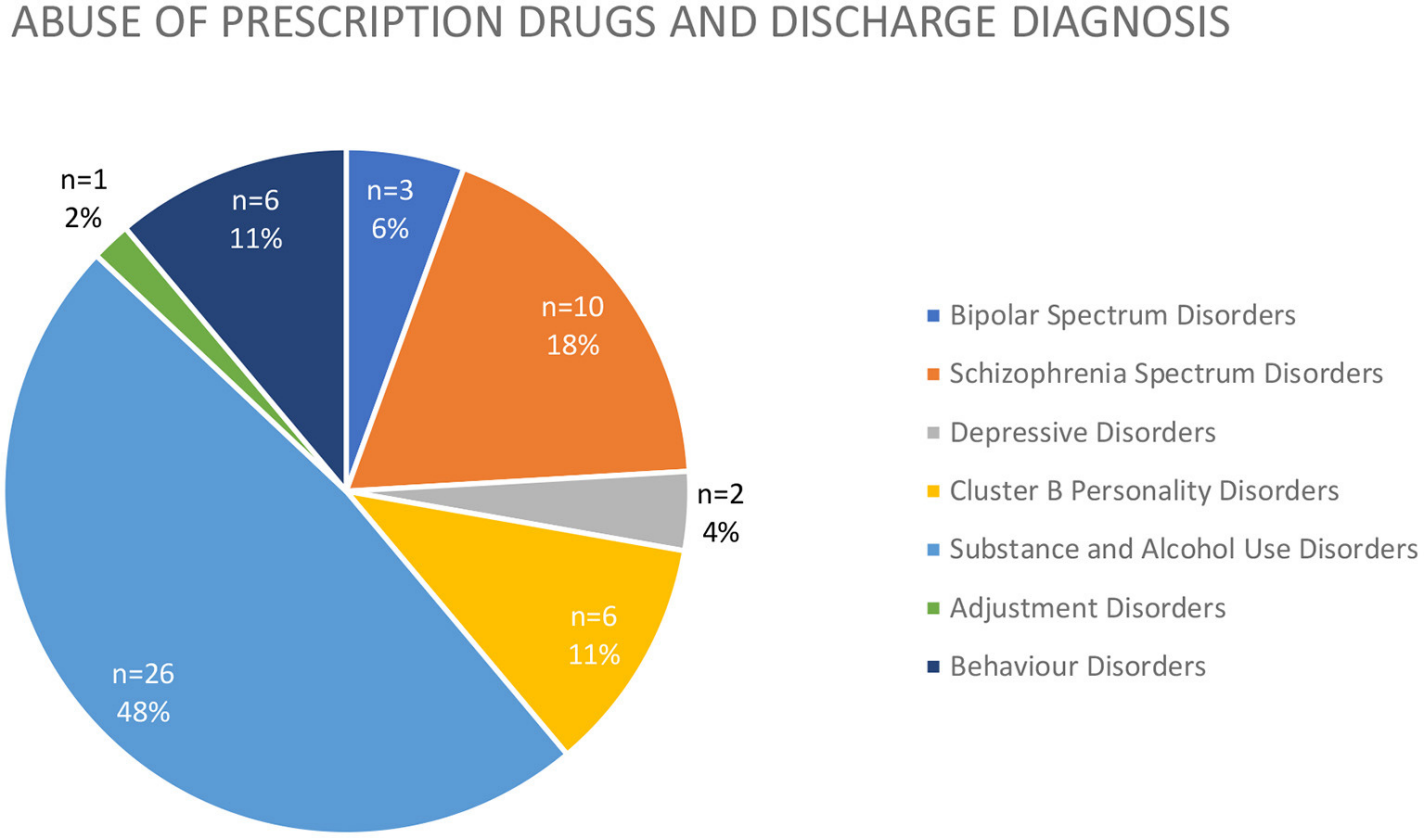
Discharge diagnosis (expressed both as raw number and %) of patients abusing prescription drugs.

The most common diagnosis at discharge among the patients who disclosed prescription drug use was substance or alcohol use disorder (*n* = 26, 48%), followed by schizophrenia spectrum disorders (*n* = 10, 18%) (Figure 4).

## Discussion

Our study evaluated the use of prescription drugs among a sample of clubbers, who were mainly composed of young subjects (more than 50% of the participants being aged under 30) with a medium-high socioeconomic status. Many subjects (40%) reported the use of prescription drugs. Therefore, our results show that such use is not only limited to subjects with psychiatric disorders and co-occurring SUD but can also involve subjects who are usually not considered as typical psychoactive substance users. This data pave the way for serious considerations on the possible pharmacological interactions with alcohol and other substances, as well as on other short- and long-term consequences, both physical and psychiatric. As users may concomitantly consume various prescription drugs and substances of abuse, an increased risk of drug–drug interactions may be observed, both pharmacokinetic (e.g., between prescription opioids and heroin) and pharmacodynamic (e.g., between opioids of abuse and benzodiazepines or other CNS sedative drugs) (41). This involves not only depressors, such as benzodiazepines, opioids, and alcohol, but also stimulant drugs commonly used by clubbers. For example, metabolic pathways of synthetic cathinones, antidepressants, and ADHD medications have been shown to overlap, including metabolism via cytochrome P450 enzymes and their inhibition (42).

Benzodiazepines were the most prevalent class of prescription drugs reported in our sample. This result may be explained by the use of benzodiazepines as a “trip terminator” to calm down the strong experience caused by the use of multiple substances. This confirms the data from Messina et al. (10), who showed that benzodiazepines and atypical antipsychotics are often used by club goers to counteract the effects of psychostimulant drugs, such as cocaine or MDMA. In terms of preventive strategies, the use of benzodiazepines in the context of a multiple substance use could be dangerous as it causes respiratory depression and risk of overdoses, specifically in combination with opiates, alcohol, ketamine and derivatives, and inhalants (18, 43, 44). Specific policies and harm-reduction approaches should be advised for these potentially lethal combinations, particularly with the intake of large amounts of long half-life compounds, such as diazepam. Furthermore, a number of novel designer benzodiazepines, with undisclosed toxicological profiles and variable potencies, have recently been made available in the drug market. They are developed in order to mimic prescription benzodiazepines and Z-drugs, but they may lead users to adverse events of various severities, particularly if used in combination with other substances (4, 45, 46).

Among the different categories of substances, psychodepressors were the most commonly associated with the use of prescription drugs, whereas only a small percentage of psychodysleptic users reported such habit. The typology of subject using psychodysleptics such as LSD, psilocybin, MDMA, ayahuasca, and other plants, is characterized by the search for a strong inner experience, spirituality, and high level of emotionality (47, 48). The use of benzodiazepines and antipsychotics can inhibit or temper the perception of these experiences and therefore may not be chosen by users. With regard to antidepressants, which can determine affective blunting and enhance the distance from emotional experiences, the same consideration can be reported.

Interestingly, patients who disclosed prescription drug misuse tended to report higher scores in HAM-D and HAM-A Somatic Anxiety. This finding emphasizes how those patients are the most vulnerable in terms of psychopathological load. In this regard, those who report taking prescription drugs may actually be the subjects with a psychiatric history. A prescription drug may have already been tested for therapeutic purposes and therefore may have made the patient more accustomed to its use out of indication. Moreover, the high level of depression is an issue that needs to be considered and can represent a significant suicidal risk factor in people who misuse alcohol and psychoactive substances. In fact, the use of psychotropics can represent an additional risk factor, given the possibility of a consistent increase in the levels of impulsivity, violence, and self-directed aggression due to such drugs. Therefore, it is very relevant to evaluate these patients and to put specific strategies in place to manage these psychopathological manifestations, with a specific focus on the prevention of anti-conservative behaviors.

A further point of interest, although expected, is the presence of high levels of depressive symptoms on the Hamilton scale in relation to the use of antidepressants without a specific medical prescription. This fact suggests how sometimes the use of prescription drugs may be related not only to the goal to “get high” or to the management of an intoxication but also to the self-medication need of patients who perceive a sub-leveling of their mood. For this reason, a shared strategy could be justified, even more than in other types of patients with dual disorders. Conversely, methylphenidate use was associated with lower scores at the Hamilton depression scale. This prescription drug with stimulant properties (49, 50), usually indicated for attention deficit hyperactivity disorder, can probably be chosen by users of psychostimulants as a cheaper alternative to cocaine and amphetamine. In the short run, it could also show some antidepressant properties, thus explaining the data observed at the HAM-D. The detection of methylphenidate among the prescription drugs reported in our sample may indicate some level of comorbidity between adult ADHD and SUD, as recently reported (51).

In terms of the role of the discharge psychiatric diagnosis, alcohol or substance use disorder showed a high prevalence, although the diagnoses of schizophrenia and bipolar spectrum disorder were also significantly reported. In some cases, the presence of a psychiatric comorbidity could justify the use of prescription drugs such as antidepressants, mood stabilizers, and benzodiazepines. However, the presence of a relevant percentage of addiction diagnoses (alcohol use disorder and/or substance use disorder) further confirms that these patients do not typically represent pure psychiatric patients who increase their dosages of prescribed drugs but are instead classical party-goers who use prescription drugs for other purposes.

Limitations of this study are represented by a low and heterogeneous sample size, with a high prevalence of benzodiazepine as the main prescription drug. Moreover, although the target of the study is that of young clubbers, a significant subgroup of participants were middle-aged adults.

In conclusion, in this study, we have highlighted how the use of prescription drugs is common also among clubbers and disco-goers. These subjects usually do not have a previous psychiatric history and share a low propensity to be prescribed with benzodiazepines, antipsychotics, and antidepressants by a mental health professional. These data confirm that prescription drugs may be an alternative for classic and novel psychoactive compounds, may be used to modulate and temper the experience, and, in some cases, may be used to reduce the negative effects determined by the use of substances. From the treatment prospective and as a useful preventive strategy, a specific psycho-education process should be indicated for subjects at risk. Party-goers should be adequately informed about the possible risks of co-intake of NPS, classical substances, and prescription drugs to prevent serious medical and psychiatric consequences.

## Data Availability Statement

The raw data supporting the conclusions of this article will be made available by the authors, without undue reservation.

## Ethics Statement

The studies involving human participants were reviewed and approved by University of Hertfordshire CEI Illes Balears University G. d'Annunzio of Chieti-Pescara. The patients/participants provided their written informed consent to participate in this study.

## Author Contributions

MdG and AN wrote the manuscript. CV, JL, MC, CD, AN, and GM recruited patients inside the Can Misses Hospital of Ibiza. SS and LT performed the statistical analysis. MP, VV, FS, and GM elaborated the study protocol and performed the translation for scales and questionnaire. FD-G, RG, CC, and MM executed the urine analysis in the different centers. AB and DD performed literature search about the topic and elaborated all the ethical procedures required for the study approval in both countries. GM coordinated all the study processes. All authors contributed to the article and approved the submitted version.

## Conflict of Interest

The authors declare that the research was conducted in the absence of any commercial or financial relationships that could be construed as a potential conflict of interest.
